# Editorial: Neurotransmitters and Emotions

**DOI:** 10.3389/fpsyg.2020.00021

**Published:** 2020-01-29

**Authors:** Fushun Wang, Jiongjiong Yang, Fang Pan, Roger C. Ho, Jason H. Huang

**Affiliations:** ^1^Institute of Brain and Psychological Science, Sichuan Normal University, Chengdu, China; ^2^Department of Psychology, Beijing University, Beijing, China; ^3^Department of Psychology, Shandong University, Jinan, China; ^4^Department of Psychology, National University of Singapore, Singapore, Singapore; ^5^Department of Neurosurgery, Baylor Scott & White Health Center, Temple, TX, United States; ^6^Department of Surgery, Texas A&M University, Temple, TX, United States

**Keywords:** neurotransmitters, emotions, dopamine, monoamine, norepinephrine

Despite the central importance of emotions for human existence (LeDoux, 1995, 2000), many debates are still had over the definition of emotion, the number of discrete basic emotions that exists, and whether different emotions have different physiological signatures (Gu et al., 2015). Although there has been no shortage of psychological research on these matters involving emotions, many core issues remain unaddressed (Gu et al., 2016). However, discoveries on representations of emotions in the brain may shed light on the nature of the complex emotional processes. In the efforts of elucidating the neural basis of emotions, the majority of the work focused on identifying neural structures responsible for the experience of particular emotions, which culminated in the limbic system theory of emotion in the mid-twentieth century. This approach to emotions has outlined the neural anatomical basis of emotions, and located important structures involved in basic emotion (LeDoux, 2000). This is consistent with the proposal of Basic Emotion Theory, which suggests that every basic emotion has a specific brain locus. This work was demonstrated by fMRI studies. However, many recent studies revealed that multiple neural structures could be implicated in one particular basic emotion, while a specific area could attribute to a number of basic emotions. For example, the amygdala has been recognized as the central site for all negative emotions, including fear and anger (Gu et al., 2019). In all, inconsistent findings have invoked numerous disputations on the neural basis approach to the study of basic emotions (Lindquist et al., 2012, 2013; Gu et al., 2019).

Here, we introduce an alternative approach—neuromodulators—to the study of emotions. Instead of isolated small brain areas, we hypothesize that basic emotions derive from the widely projected neuromodulators, such as dopamine (DA), serotonin (5-HT), and norepinephrine (NE). Darwin proposed that phylogenetically lower animals, such as insects, also have basic emotions, but they have distinctly different brain structures with similar monoamine neuromodulators. Ever since its discovery, monoamine has been deemed as the substrate for emotions. Antidepressants affecting monoamine neuromodulators have been used for almost all affective disorders (Lovheim, 2012; Lohoff et al., 2014). Even decades later, monoamine-targeted drugs are still the first-line of pharmacological treatment for affective disorders, such as anxiety, phobia, and depression (Gu et al., 2018b). Human eyes are able to perceive all colors thanks to the three types of cone cells, with each type sensitive to one of the three primary colors. Similar to the perception of color, we herein propose a new theory of emotion—“three primary color model” of basic emotions (Figure 1) (Gu et al., 2018a, 2019)”—that emotion is a product from the mixture of the three monoamines (Lovheim, 2012; Gu et al., 2018a). This theory of emotion states that central DA is a hedonic signal for salient stimuli, such as food, sex, and other needs; central 5-HT is related to disgust or punishment; and central NE is the substrate for emotions that trigger “fight or flight” response, such as fear and anger (Gu et al., 2019). Many recent emotion experimental and theoretical studies surging up recently support this emotion theory based on the three monoamines (Lovheim, 2012).

**Figure 1:**
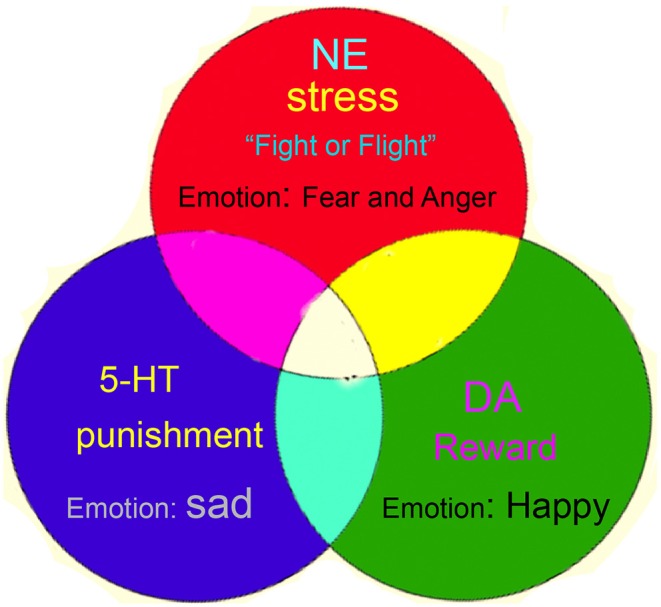
Monoamine model of basic emotions. The number of basic emotions we humans have is still controversial. We propose a monoamine model of basic emotions, or called “three primary color model” of basic emotions. Norepinephrine (NE) is responsible for fear and anger emotions that trigger “fight or flight” response; fear and anger are classified as one core emotion—the stressful emotion—like two sides of the same coin. Joy is subsided by dopamine (DA), while punishment is subsided by serotonin (5-HT) (Adopted from our previous paper: Gu et al. Frontiers in Psychology, 2018, 9:1924).

In addition to these monoamines, other hormones and neuromodulators may also be involved in a secondary pathway. For example, corticotropin-releasing hormone (CRH)—the stress hormone—might be involved in a pathway of central norepinephrine (NE) release (Simeon et al., 2007). The CRH induces the release of ACTH (adrenocorticotropic hormone), which can, in turn, alters physiological processes and behaviors (Chauhan et al., 2017). Ketamine, once known as a blocker to a particular glutamate receptor, has recently been used as an antidepressant (Yang et al., 2018). Oxytocin is believed to be related with love and attachment (Aguilar-Raab et al., 2019). Recent studies have proven that many neurotransmitters may play a pivotal role in emotions as substrates for emotions. Simply put, *emotions are nothing but neuromodulators*. We have solicited the most advanced studies evaluating the emotional functions of neurotransmitters, and accepted 11 peer-reviewed papers in this special collection.

Liu et al. introduced a literature review that probed into the relationship between the dysfunction of serotoninergic, noradrenergic, and dopaminergic systems with major depression disorders in multiple ways, such as animal studies and human imaging studies. They found that the altered monoamine neurotransmitter functions play a critical role in the mechanism of emotional disorders.

Gu et al. introduced three core affects: happiness, sadness, and stress, which are subsided respectively by three neuromodulators: dopamine, serotonin, and norepinephrine. Complex emotions are analogous to colors in the way that they are results of a proportional mix of the three core affects.

Ye et al. tried to relate the emotional neurotransmitters to the mechanism of Traditional Chinese Medicine.

Langenecker et al. presented genetic analysis with the serotonin transporter and monoamine oxidase A genes. Interestingly, they found that higher serotonin1A binding potentials were related to a substantial memory bias toward negative emotions.

Bian et al. identified several genes involved in post-traumatic stress disorders.

Shi et al. found that the dopaminergic reward system is impaired in bipolar depressive patients.

Yang et al. found that hydrogen sulfide in hippocampus might affect neurotransmitters to modulate sleep deprivation induced deficit of cognition.

Yu et al. investigated the neurophysiological characteristics of young people with depressive personality disorders.

Wei J. et al. reported their studies about the brain network involved in the development of blepharospasm, with a particular focus on inferior frontal gyrus, posterior cingulate cortex, and temporal gyrus.

Meng et al. reported a significant interaction effects of childhood maltreatment and emotion on executive attention scores in reaction times that reflect conflict resolution speed. They concluded that childhood maltreatment can induce brain dysfunctions, which are sensitive to negative emotional stimuli.

Wei S. et al. reported their studies on gene expressions in the hippocampus related with aggressive behaviors of premenstrual dysphoric disorders.

Collectively, these studies strengthen the association between neurotransmitters and basic emotions. Owing to the intricate nature of emotions, studies aiming at its connection with neurotransmitters are necessarily complex and multifocal. We sincerely hope that you will enjoy reading all the papers in this special edition.

## Author Contributions

FW, JY, FP, RH, and JH all helped in writing the editorial.

### Conflict of Interest

The authors declare that the research was conducted in the absence of any commercial or financial relationships that could be construed as a potential conflict of interest.

## References

[B1] Aguilar-Raab C. Eckstein M. Geracitano S. Prevost M. Gold I. Markus H. (2019). Oxytocin modulates the cognitive appraisal of the own and others close intimate relationship. Front. Neurosci. 13:714 10.3389/fnins.2019.00714 31379475 PMC6646594

[B2] Chauhan N. R. Kapoor M. Prabha Singh L. Gupta R. K. Chand Meena R. Tulsawani R.. (2017). Heat stress-induced neuroinflammation and aberration in monoamine levels in hypothalamus are associated with temperature dysregulation. Neuroscience 358, 79–92. 10.1016/j.neuroscience.2017.06.023 28663093

[B3] Gu S. Gao M. Yan Y. Wang F. Tang Y. Y. Huang J. H. (2018a). The neural mechanism underlying cognitive and emotional processes in creativity. Front. Psychol. 9:1924. 10.3389/fpsyg.2018.01924 30429805 PMC6220028

[B4] Gu S. Jing L. Li Y. Huang J. H. Wang F. (2018b). Stress induced hormone and neuromodulator changes in menopausal depressive rats. Front. Psychiatry 9:253. 10.3389/fpsyt.2018.00253 29951006 PMC6008427

[B5] Gu S. Wang F. Cao C. Wu E. Tang Y. Y. Huang J. H. (2019). An integrative way for studying neural basis of basic emotions with fMRI. Front. Neurosci. 13:628. 10.3389/fnins.2019.00628 31275107 PMC6593191

[B6] Gu S. Wang F. Yuan T. Guo B. Huang J. H. (2015). Differentiation of primary emotions through neuromodulators: review of literature. Int. J. Neurol. Res. 1, 43–50. 10.17554/j.issn.2313-5611.2015.01.19

[B7] Gu S. Wang W. Wang F. Huang J. H. (2016). Neuromodulator and emotion biomarker for stress induced mental disorders. Neural Plast. 2016:2609128. 10.1155/2016/2609128 27051536 PMC4808661

[B8] LeDoux J. E. (1995). Emotion: clues from the brain. Annu. Rev. Psychol. 46, 209–235. 10.1146/annurev.ps.46.020195.001233 7872730

[B9] LeDoux J. E. (2000). Emotion circuits in the brain. Annu. Rev. Neurosci. 23, 155–184. 10.1146/annurev.neuro.23.1.155 10845062

[B10] Lindquist K. Wager T. Kober H. Bliss-Moreau E. Barrett L. (2012). The brain basis of emotion: a meta-analytic review. Behav. Brain Sci. 35, 121–143. 10.1017/S0140525X11000446 22617651 PMC4329228

[B11] Lindquist K. A. Siegel E. H. Quigley K. S. Barrett L. F. (2013). The hundred-year emotion war: are emotions natural kinds or psychological constructions? Comment on Lench, Flores, and Bench (2011). Psychol. Bull. 139, 255–263. 10.1037/a0029038 23294094 PMC3556454

[B12] Lohoff F. W. Hodge R. Narasimhan S. Nall A. Ferraro T. N. Mickey B. J.. (2014). Functional genetic variants in the vesicular monoamine transporter 1 modulate emotion processing. Mol. Psychiatry 19, 129–139. 10.1038/mp.2012.193 23337945 PMC4311877

[B13] Lovheim H. (2012). A new three-dimensional model for emotions and monoamine neurotransmitters. Med. Hypotheses 78, 341–348. 10.1016/j.mehy.2011.11.016 22153577

[B14] Simeon D. Knutelska M. Smith L. Baker B. R. Hollander E. (2007). A preliminary study of cortisol and norepinephrine reactivity to psychosocial stress in borderline personality disorder with high and low dissociation. Psychiatry Res. 149, 177–184. 10.1016/j.psychres.2005.11.014 17169436

[B15] Yang Y. Cui Y. Sang K. Dong Y. Ni Z. Ma S.. (2018). Ketamine blocks bursting in the lateral habenula to rapidly relieve depression. Nature 554, 317–322. 10.1038/nature25509 29446381

